# Supplementary material for: Analysis of Co-Crystallization Mechanism of Theophylline and Citric Acid from Raman Investigations in Pseudo Polymorphic Forms Obtained by Different Synthesis Methods
Source: Molecules. 2023 Feb 7;28(4):1605. doi: 10.3390/molecules28041605 (PMC9960353; doi:10.3390/molecules28041605)
Supplement: Supplementary file 1 [file molecules-28-01605-s001.zip › molecules-2110698-supplementary.pdf]

Supplementary Materials

Analysis of Co-Crystallization Mechanism of Theophylline and Citric Acid from Raman Investigations in Pseudo Polymorphic Forms Obtained by Different Synthesis Methods

Yannick Guinet, Laurent Paccou and Alain Hédoux

Univ. Lille, CNRS, INRAE, Centrale Lille, UMR 8207 - UMET - Unité Matériaux et Transformations, F-59000 Lille, France

Figure S1. Fitting procedure of the low-frequency spectrum in co-crystals

Figure S2. Temperature dependence of the O – H stretching band in the anhydrous co-crystal

Figure S3. Temperature dependence of the high frequency spectrum taken in the co-crystal hydrate

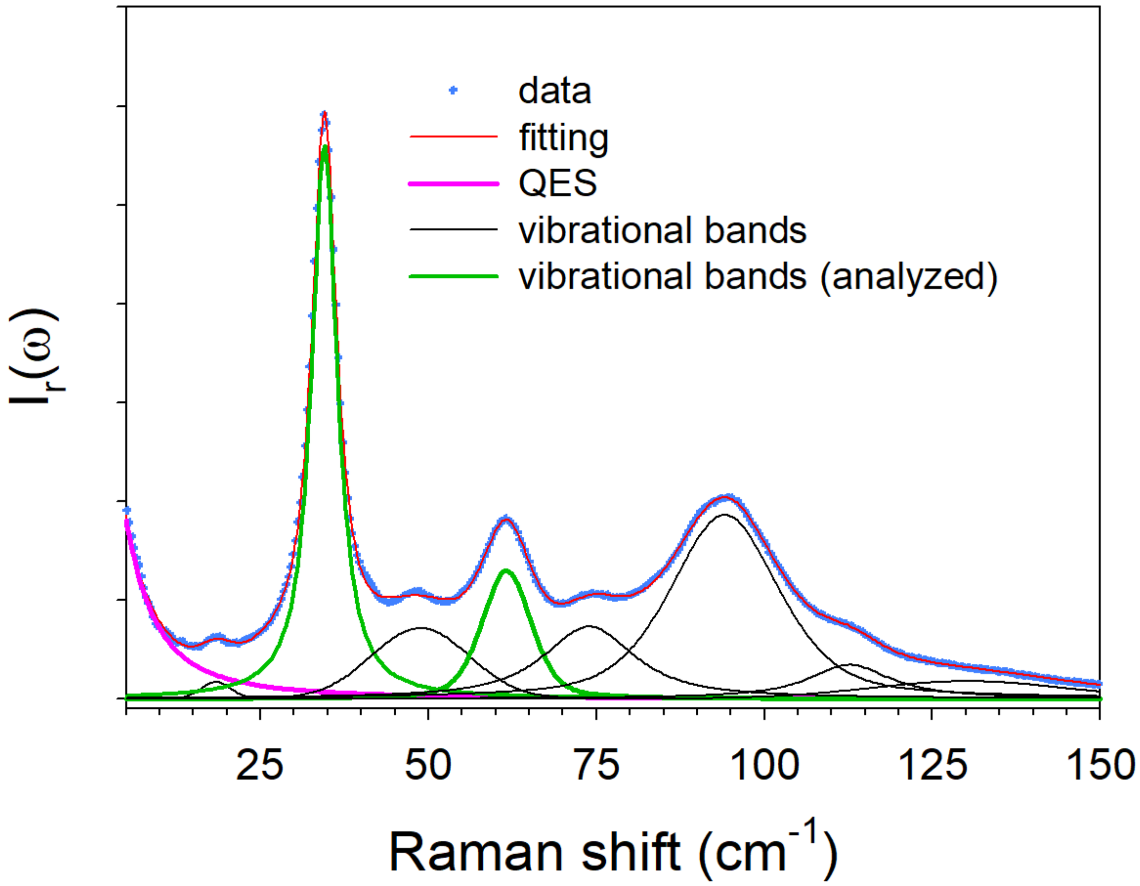

Figure S1. Fitting procedure of the low-frequency spectrum in co-crystals. Bands plotted in **green thick lines** and the quasielastic scattering (QES) have been analyzed in anhydrous and hydrate forms.

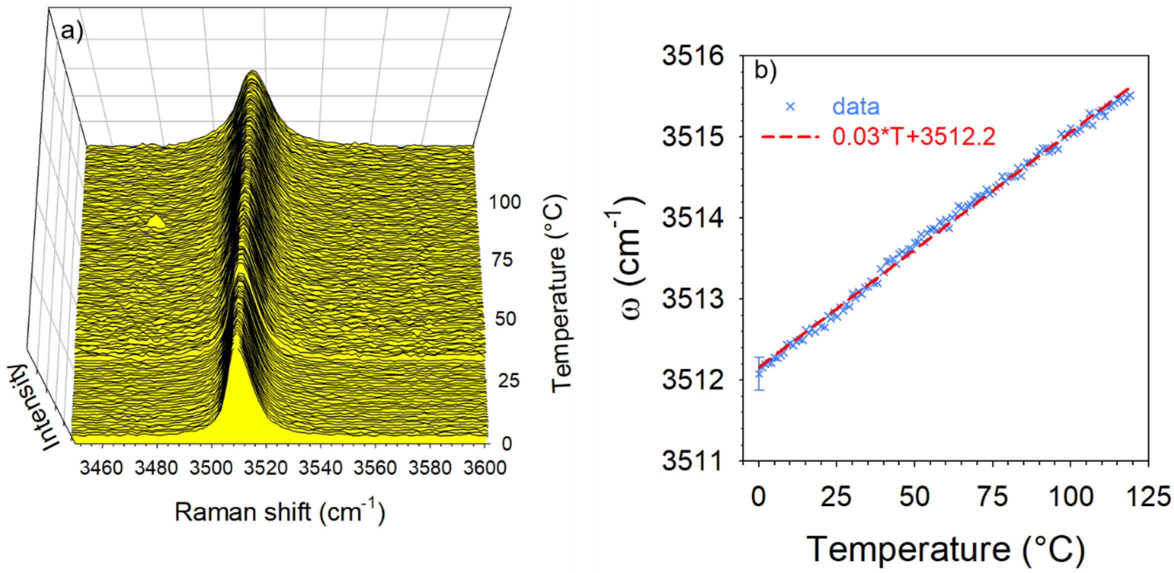

Figure S2. Temperature dependence of the O – H stretching band in the anhydrous co-crystal, typical of vibrations of OH groups within citric acid molecules involved in π-H-bonding with TP molecules; **a**) temperature dependence of the spectrum; **b**) temperature dependence of the position of the O – H stretching band obtained by fitting the band with mixed Gaussian-Lorentzian peak – the dashed line corresponds to the fit of data with a linear regression.

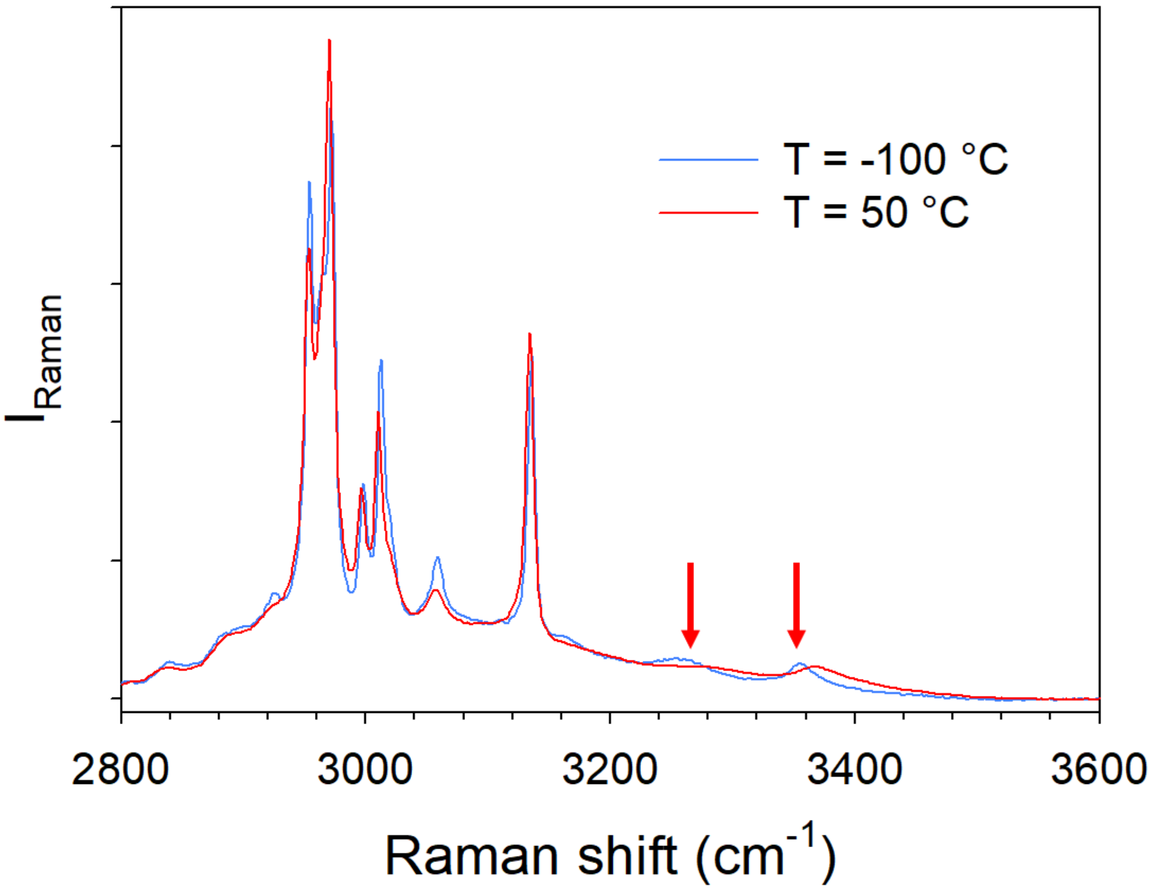

Figure S3. Temperature dependence of the high frequency spectrum taken in the co-crystal hydrate. The arrows show the two O – H stretching bands which exhibit a signature of H-bonding, i.e. a positive temperature dependence of the position. These bands have assigned to vibrations of OH groups within water molecules bound interacting with TP and CA molecules
